# Helpful viability of post calving tonic for the treatment of post calving complications in dairy animals

**DOI:** 10.5455/javar.2021.h557

**Published:** 2021-11-06

**Authors:** Manu Kumarswamy, Vishwanath Gopal Bhagwat, Santoshkumar Tattimani, Rangesh Paramesh

**Affiliations:** 1 Veterinary Consultant, Nelamangala, India; 2 Animal Health Division, Research and Development Center, Himalaya Wellness Company, Makali, India

**Keywords:** Dairy, bovines, ecbolic, uterine secretions, milk yield

## Abstract

**Objectives:**

Aimless use of anti-microbials for the treatment of uterine diseases has driven the rise of safe strains. Subsequently, within the current consider, the viability of post calving tonic (PCT) was assessed *in vivo* for the treatment of post calving complications in dairy cattle.

**Materials and Methods:**

The placentas of 10 chosen post-calved dairy animals with a history of postpartum complications primarily held placentas were drenched with PCT 250 ml twice a day for 2 successive days. The evaluation parameters, *viz*., time taken for removal of lochia, placenta, involution of the uterus and estrus come back time, body condition score, and milk yield have been analyzed to assess the efficacy of PCT in post calved dairy bovines.

**Results:**

The results depicted that following the administration of PCT, the mean time taken for expulsion of uterine discharge (lochia), placenta, time taken for the involution of the uterus, and estrus come back time was 86 and 10 h, 30 and 36 days, respectively. Additionally, the administration of PCT 250 ml twice daily for 2 consecutive days to the post-calved dairy cows caused a significant increase (*p* < 0.05) in milk yield.

**Conclusion:**

It was apparent that PCT encourages the unconstrained removal of held placenta through its ecbolic movement and advances uterine discharge exercises. In addition, PCT supplementation caused augmentation of milk yield in post calved dairy cows.

## Introduction

The postpartum period is the most important transitional period in cattle life, during which various physiological, gynecological, and biochemical changes occur. During this time, the anatomical barrier is breached, and the genitals remain open for several days, increasing the risk of infection in the womb [1]. Therefore, dairy cows usually experience reproductive problems during and after calving. After calving, reproductive efficiency is restored by resuming normal ovarian activity and uterine involution. The constriction of the uterus, detachment of the caruncle, and regeneration of the endometrium are all stages of uterine involution [2]. When the uterus is reduced to its pre-pregnancy size, complete uterine involution is observed [3].

The volume of endometrial fluid, as evaluated by endometrial area recovery, has been employed as a uterine normality indicator [4,5]. After calving, the first ovarian cycle is shorter than a cow’s usual estrous cycle. Ovulation occurs 17–34 days following the first calving in the majority of cows [6]. Cows milked twice daily experience their first ovulation 19 ± 1 days after calving, with the earliest ovulation occurring 10 to 15 days after calving [7]. Dairy cow calving may be improved by early postpartum ovulation and pregnancy during the three estrous cycles [8,9]. The return of ovarian activity is indicated by the emergence of the first considerable increase in progesterone (1 ng/ml) [10].

In most cows, uterine involution is completed in the first 4–5 weeks after calving. The earliest period of uterine involution is about 3 weeks after delivery. If the fetal membrane is preserved and/or endometritis develops in the bovine uterus, the time to involution of the uterus can be 30–50 days longer [11]. During the usual involution process, the liquid medium (lochia) produces large amounts of necrotic tissue, which can contaminate the uterine cavity with bacteria after calving. In the healthiest cows, bacteria can be naturally removed from the uterus in the first few weeks after calf birth [12].

Retention of placenta (ROP) is one of the most common postpartum diseases of cattle. This can also lead to an increased incidence of endometritis, which reduces milk production and impairs fertility [13]. In addition, uterine infections tend to increase herd health costs, reduce feed consumption, and increase culling rates [14]. Also, lower fertility rates in dairy cows are one of the most important factors affecting farm profitability [15].

The ideal treatment goal for retained fetal membranes is to promote placental detachment and its excretion from the uterine cavity, eliminating bacterial contamination of the uterus. Although recommended at various times, oxytocin, estradiol, prostaglandin F2alpha, and oral calcium supplements have not been shown to accelerate the elimination of retained membranes or prevent complications [16]. If the placenta is not separated from the caruncle, oxytocin does not accelerate its passage [17]. If dairy cows are heavily infected with bacteria, they can be treated with antibiotics or hormone-based drugs. Lutein has been used to treat endometritis and uterine infections in numerous studies [18–20]. However, the concern is the issue of drug retention and tolerance. Therefore, researchers turned their attention to herbal preparations [21]. In addition, the literature reports show that various folk veterinary materials reported in such as *Vitex doniana* (bark), *Hibiscus esculentus* (fruit), *Carica papaya* (leaves), *Salvadora persica* (root) [22], *Tribulus terrestris* L. (whole plant) [23], *Hedera helix* L. (leaves) [24], *Debra glabra (*leaves) and *Dobera loranthifolia* (leaves) [25], *Aloe tenuior* Haw (Leaf) [26] and *Glyphaea brevis* (leaf), and *Spondias mombin* (leaf) [27] are used to eliminate ROP in cattle. 

With the background of the growing acceptance of traditional herbal preparations, in the present study, post calving tonic (PCT), a polyherbal formulation developed by the Himalaya Wellness Company, was evaluated for its effectiveness in the treatment of postnatal complications in dairy cattle has been studied.

## Materials and Methods

### Ethical committee approval

This study was conducted in accordance with guidelines formulated for animal care and use and the study protocol was approved by the Institutional Animal Ethics Committee, Himalaya Wellness Company, Bangalore, Protocol No. AHP/LA/11/19.

### Poly herbal formulation

PCT was previously HimROP® Plus Vet Liquid and is a proprietary poly herbal formulation developed by the Himalaya Wellness Company in Bengaluru, India. PCT is composed of leaves of *C. papaya*, *Moringa oleifera*, tuber parts of *Gloriosa superba*, *Cyperus rotundus*, aerial parts of *Adhatoda vasica*, and seeds of *Peganum harmala.*

### Study subjects

A total of 10 post calved dairy cows of Holstein Friesian and Jersey cross breeds aged between 4 and 5 years and parity between 1 and 3 at Nelamangala area, Bangalore Rural, Karnataka were selected. Furthermore, dairy cows with a history of ROP, uterine infections, delayed uterine involutions, poor uterine tonicity, repeat breeding, and post calving complications due to energy deficit were selected. The cows were used as their own controls and, therefore, were allocated to a control pretreatment period (0 day), followed by a treatment period (2 days). The study excluded cows with no gag reflex and severe disease conditions such as bovine tuberculosis and prolapse.

### Study design and experimental details

A total of 10 selected post calved dairy cows with a history of ROP and other postpartum complications G1 (*n* = 10) were drenched with PCT twice a day for 2 consecutive days. PCT dosage was selected as per the label recommendations and was also the intended dose for the target species for specified indications. When PCT was administered to the dairy cows, concurrent treatment with other herbal-based products was not followed.

### Evaluation of study parameters

The parameters of evaluation, the time required for the expulsion of the lochi, the placenta, the involution of the uterus and the return time of estrus, the body condition score, and milk production to evaluate the effectiveness of PCT in postpartum dairy cows.

### Statistical analysis

Data are expressed as mean ± standard error of mean (SEM). The body condition score was subjected to a paired *t*-test and the milk production data was subjected to repeated measurements, one-way analysis of variance, followed by Dunnett’s *post-hoc* multiple comparison test to assess the impact of PCT in milk production between week 1 of supplementation and the following weeks, i.e., week 2 and 3 weeks of integration. A *p* value ≤ 0.05 was considered as statistically significant.

**Figure 1.**
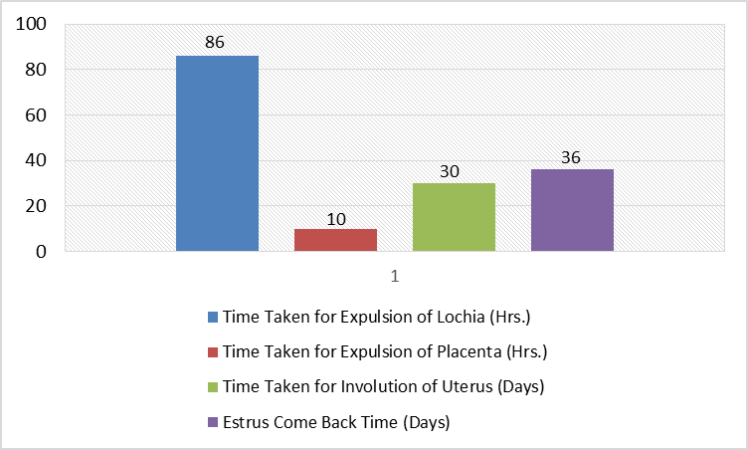
Effect of PCT on time taken for lochia expulsion, time taken for expulsion of placenta, uterus involution time, and estrus come back time.

**Figure 2.**
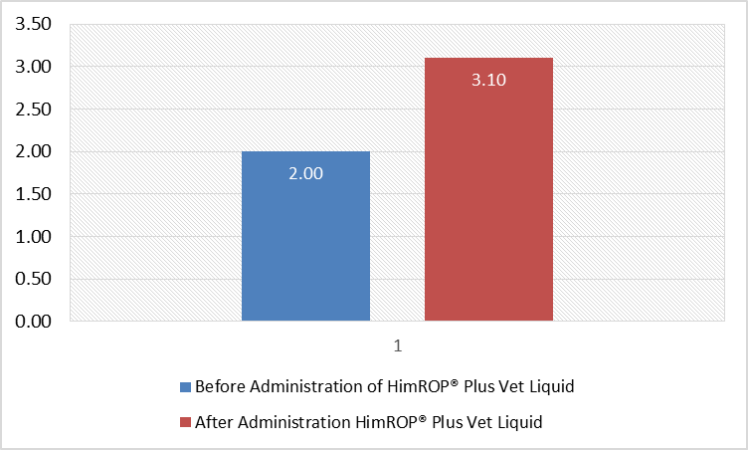
Effect of PCT on body condition score of dairy cows. ****p < 0.0001 as compared to before administration of PCT based on paired *t*-test.

## Results and Discussion

After administration of PCT, the mean time required for the expulsion of uterine secretion (lochia), placenta, involution of the uterus, and estrus come back time was 86 and 10 h, 30 and 36 days, respectively (Fig. 1). A statistically significant (*p* < 0.0001) improvement in the body condition score of dairy cows was observed following PCT administration (Fig. 2). Additionally, following PCT administration of 250 ml twice daily for 2 consecutive days to the post-calved dairy cows caused an improvement in the sum milk yield and a significant (*p* < 0.05) improvement in the mean milk yield as compared to that in week 1 (Table 1).

Manual removal is the oldest and most common method of treatment, though not completely satisfactory. The use of collagenase may aid manual removal as it facilitates placental separation [28]. One of the major preventive measures to check post-parturient reproductive failure is the care and follow-up of animals that have had ROP, abnormal discharge, postpartum anestrus, or endometritis. ROP is a very common sequela. Antibiotics and estrogens have been used to treat ROP conditions but are not routinely effective or free from deleterious side effects. Various other treatments have been tried, including ergot, oxytocin, and estrogen, but they are not satisfactory [29]. Therefore, oral administration of herbs with proven uterine and ecbolic tonics and cleansing effects appears to be a safe and effective option, both therapeutically and prophylactically. Therefore, the current study was carried out to evaluate the effectiveness of PCT in treating postpartum complications in dairy cows.

**Table 1.** Impact of PCT on sum milk yield in dairy cows.

Sl	Parameter	Week 1 (average of days 1–7)	Week 2 (average of days 8–14)	Week 3 (average of days 15–21 average)
1	Sum milk yield (l)	139.64	155.10	162.93
2	Mean milk yield (l)	13.96 ± 0.96	15.51 ± 0.48	*16.29 ± 0.59

Values are expressed as mean ± SEM; *n* = 10.

* *p *< 0.05 (Significantly increased) compared to week 1 values based on repeated measures of one-way analysis of variance followed by Dunnett’s Multiple Comparison *post hoc* test.

In our study, the time taken for the expulsion of lochia and placenta following PCT administration in dairy cows presented with post calved complications was 86 and 10 h, respectively. These findings delineated that oral administration of PCT effectively facilitated the spontaneous expulsion of the placenta; this could be due to its herbal ingredients, mainly including *C. papaya*,* G. superba*, and* P. harmala* present in PCT that could provide a more favorable environment for placenta detachment through their ecbolic activities and uterine cleansing action through activation of uterine secretions. Dharani et al. [30] have shown that the leaves of *C. papaya* in combination with the roots of *Harrisonia abyssinica*, *Grewia villosa* and *Ricinus communis*, and the barks of *Acacia drepanolobium* are useful in expelling the ROP from cows. Furthermore, according to literature reports, trials showed that *G. superba* and *P. harmala* were used as one of the main ingredients in the herbal formulation due to their potential ecbolic activity [31,32].

Literature reports show that during the last 20 years, *A. vasica *and alkaloids derived from the plant have mimicked the biological activities of oxytocic and abortifacient effects [33]. In our study, the results of time taken for the involution of the uterus and estrus come back time following PCT administration to dairy cows presented with post calving complications depicted that uterine involution for dairy cows takes an average of 30 days and estrus come back takes 36 days that falls within the normal range. These findings could be attributed to *A. vasica *present in PCT that could promote the myometrial contractility after calving, can increase the contractile activity of the uterus, and can also help the uterus return to its antiverse position.

Several studies published by various researchers in the literature have revealed that supplementation with *M. oleifera* leaves plays a crucial role in increasing milk production. Cohen-Zinder et al. [34] reported a significant increase in milk production, milk fat, and protein content in lactating cows after supplementing with a diet containing *M. oleifera* leaves. Another research study by Azzaz et al. [35] found that the increase in milk production and total solids was 11.3% and 17.7% in lactating sheep, followed by supplementation with a diet containing *M. oleifera* leaves. This favorable effect of *M. oleifera* on cattle production performance could be due to improved feed intake, apparent nutrient digestibility, and rumen fermentation conditions [34,36]. Concurrent with literature findings, administration of PCT 250 ml twice daily for two consecutive days to the post-calved dairy cows caused a significant (*p* < 0.001) increase in the milk yield, and this could be attributed mainly to the leaves of *M. oleifera *present in PCT.

## Conclusion

In conclusion, it was apparent from the present study that PCT is an elite combination of standardized and exceedingly useful therapeutic herbs with a strong ecbolic movement and viably encourages the unconstrained ejection of held placenta. Additionally, PCT actuates a successful uterine cleansing activity through the actuation of uterine discharges. Besides, PCT seems to play a significant part in the stipend of financial misfortunes due to ROP complications through milk yield augmentation. 

## List of Abbreviations

d, Day; h, Hour; ml, Milliliter; l, liter; PCT, Post calving tonic; ROP, Retention of placenta; SEM, Standard error of mean.
